# Self-reported sexually transmitted infections and their correlates among men who have sex with men in Norway: an Internet-based cross-sectional survey

**DOI:** 10.1186/1471-2334-10-261

**Published:** 2010-09-06

**Authors:** Irena Jakopanec, Barbara Schimmer, Andrej M Grjibovski, Elise Klouman, Preben Aavitsland

**Affiliations:** 1 Department of Infectious Disease Epidemiology, Norwegian Institute of Public Health, PO Box 4404 Nydalen, N-0403 Oslo, Norway; 2 National Institute for Public Health and the Environment, Bilthoven, the Netherlands; 3 Institute of Community Medicine, University of Tromsø, Tromsø, Norway; 4 International School of Public Health, Northern State Medical University, Arkhangelsk, Russia

## Abstract

**Background:**

The incidences of reportable sexually transmitted infections (STI) among men who have sex with men (MSM) have increased since the late 1990 s in Norway. The objectives of our study were to assess factors, associated with recent selected STI among MSM, living in Norway in order to guide prevention measures.

**Methods:**

We conducted a cross-sectional Internet-based survey during 1-19 October 2007 among members of a MSM-oriented Norwegian website using an anonymous questionnaire on demographics, sexual behaviour, drug and alcohol use, and STI. The studied outcomes were gonorrhoea, syphilis, HIV or Chlamydia infection in the previous 12 months. Associations between self-reported selected STI and their correlates were analysed by multivariable Poisson regression. P value for trend (p-trend), adjusted prevalence ratios (PR) with 95% confidence intervals [] were calculated.

**Results:**

Among 2430 eligible 16-74 years old respondents, 184 (8%) reported having had one of the following: syphilis (n = 17), gonorrhoea (n = 35), HIV (n = 42) or Chlamydia (n = 126) diagnosed in the past 12 months. Reporting Chlamydia was associated with non-western background (PR 2.8 [1.4-5.7]), number of lifetime male partners (p-trend < 0.001), unsafe sex under the influence of alcohol (PR 1.8 [1.1-2.9]) and with younger age (p-trend = 0.002). Reporting gonorrhoea was associated with unrevealed background (PR 5.9 [1.3-26.3]), having more than 50 lifetime male partners (PR 4.5 [1.3-15.6]) and more than 5 partners in the past 6 months (PR 3.1 [1.1-8.8]), while mid-range income was protective (PR 0.1 [0.0-0.6]). Reporting HIV was associated with residing in Oslo or Akershus county (PR 2.3 [1.2-4.6]), non-western background (PR 5.4 [1.9-15.3]), unrevealed income (PR 10.4 [1.5-71.4]), number of lifetime male partners (p-trend < 0.001) and being under the influence of selected drugs during sex in the past 12 months (PR 5.2 [2.7-11.4]). In addition, the frequency of feeling drunk was reversibly associated with HIV.

**Conclusions:**

Our study demonstrates different associations of demographic and behavioural factors with different STI outcomes in the study population. Number of lifetime male partners was the most important potential predictor for Chlamydia and HIV. The STI prevention efforts among MSM should focus on Oslo and Akershus, promote safe sex practices and tackle sex-related drug and alcohol use.

## Background

Since the mid-1990 s, sexually transmitted infections (STI) among men who have sex with men (MSM) have been reported to be on the rise in many European countries [1] and worldwide [2,3]. Increasing numbers of HIV infections, gonorrhoea and syphilis among men, infected by other men, have been also observed in the Norwegian surveillance system for communicable diseases [4-6]. Chlamydia is the most common reportable STI in Norway [7] and although MSM-specific data are not available, the rate among Norwegian men in 2007 was as high as 368/100 000 person-years. The STI transmission mainly occurs in the capital Oslo and neighbouring Akershus county, where the MSM population is concentrated [5,8]. The surveillance system collects only limited information on STI patients and specific knowledge about sexual risk behaviour among MSM living in Norway is very limited.

The MSM population is frequently referred to as "hidden" [9,10] due to its unknown size and difficulties to reach. Internet websites have been shown to be convenient cost-effective tools for recruitment of MSM [11]. In 2007, 78% of all households in Norway had Internet access and 88% of men between 16-74 years old have used it recently [12]. Sampling on the Internet may be also more convenient to attract those men, who may be less likely to self-identify as MSM [9].

The objective of this study was to assess the associations between selected factors and self-reported STI in the past 12 months among MSM, living in Norway.

## Methods

### Participants and Recruitment

An Internet cross-sectional survey was initiated by the Norwegian Institute of Public Health, in cooperation with "Gay and Lesbian Health Norway". Participation was offered to logged-in members of the Norwegian MSM-oriented website from 1 October 2007 to 19 October 2007. This is the leading website for the MSM-net-community in the Norwegian language with more than 31 000 member profiles. The site has about 50 000 visits each week and provides news items, a chat-community, a discussion forum, an events calendar, links and other information. When clicking on the study banner, the participants were guided to an introduction, where the aims and structure of the study were explained. Anonymity of the participants was assured and contact persons for the study from collaborating institutions were provided. The participation was voluntary. Respondents, who answered that they were women, younger than 16 years, had never had sex with a man or were living abroad, were excluded from the analysis.

### Selected outcomes

We focused our analysis on MSM, who reported being diagnosed with any of the following STI in the previous 12 months: gonorrhoea, syphilis, HIV or Chlamydia infection ("selected STI"). To check if selected exposures are differently associated with a specific STI, we performed multivariable analyses for Chlamydia, gonorrhoea and HIV infection as separate outcomes (Table 1), but due to the low number of cases, this was not possible for syphilis.

**Table 1:** Overview of the multivariable models, their outcomes and comparison groups in the Internet-based cross-sectional MSM study.

Model	Outcome*	Comparison group
1.	Chlamydia, NO selected STI	respondents not reporting any selected STI

2.	gonorrhoea or gonorrhoea AND any selected STI	respondents not reporting any selected STI

3.	HIV infection or HIV infection AND any selected STI**	respondents not reporting any selected STI

* all outcomes diagnosed in the past 12 months

**selected STI - syphilis, gonorrhoea, HIV or Chlamydia in the past 12 months

### Pilot study

An offline and online testing of the questionnaire was conducted by 15 MSM, who provided a detailed feed-back on the content, functionality and the questionnaire layout.

### Data collection

Data were collected using the online survey tool Questback and harvested in Excel format.

### Measures and data presentation

Demographic characteristics and sexual behaviour were initially classified as presented in Tables 2 and 3; however some categories within variables were merged in multivariable analysis as presented in the Table 4. The variables health region and residing in Oslo/Akershus county were created from the "county of residence" variable. The variable "drugs before sex" was created based on reports of using any of the following drugs before sex: marihuana, prescription drugs, ecstasy, LSD, GHB, cocaine, heroine, amphetamines or methamphetamines.

**Table 2:** Demographic characteristics of MSM respondents in the Internet-based cross-sectional study.

Proportion of MSM, reporting Chlamydia, gonorrhoea, HIV infection or syphilis in the previous 12 months and their crude associations									
Demographic characteristics	respondents N = 2430 (%)	Chlamydia* N= 101 (%)	Crude PR Chlamydia [95% CI]	gonorrhoea N = 35 (%)	Crude PR gonorrhoea [95% CI]	HIV N = 42 (%)	Crude PR HIV [95% CI]	Syphilis N = 17 (%)	Crude PR syphilis
age groups (years)									
16-25	768 (31.6)	37 (36.6)	ref. group	16 (45.7)	ref. group	7 (16.7)	ref. group	3 (17.6)	ref. group
26-35	769 (31.6)	35 (34.6)	0.9 [0.6-1.5]	8 (22.9)	0.5 [0.2-1.2]	15 (35.7)	2.1 [0.9-5.2]	6 (35.3)	2.0 [0.5-8.0]
36-45	551 (22.7)	22 (21.8)	0.8 [0.5-1.4]	8 (22.9)	0.7 [0.3-1.6]	11 (26.2)	2.2 [0.8-5.6]	5 (29.4)	2.3 [0.6-10.0]
≥ 46	342 (14.0)	7 (6.9)	0.4 [0.2-0.9]	3 (8.6)	0.4 [0.1-1.4]	9 (21.4)	2.9 [1.1-7.7]	3 (17.6)	2.2 [0.4-11.1]

health region of residence									
East	1415 (58.2)	70 (69.3)	ref. group	26 (74.3)	ref. group	36 (85.7)	ref. group	15 (88.2)	ref. group
West	369 (15.2)	16 (15.8)	0.9 [0.5-1.5]	5 (14.3)	0.7 [0.3-1.9]	1 (2.4)	0.1 [0.0-0.8]	0	
South	254 (10.4)	7 (6.9)	0.6 [0.3-1.2]	2 (5.7)	0.4 [0.1-1.8]	2 (4.8)	0.3 [0.1-1.3]	2 (11.8)	0.7 [0.2-3.2]
Mid-Norway	236 (9.7)	7 (6.9)	0.6 [0.3-1.3]	2 (5.7)	0.5 [0.1-1.9]	1 (2.4)	0.2 [0.0-1.2]	0	
North	151 (6.2)	1 (1.0)	0.1 [0.2-0.9]	0		2 (4.8)	0.5 [0.1-2.1]	0	
unknown	5 (0.2)	0		0		0		0	

residing in Oslo/Akershus county									
no	1200 (49.4)	38 (37.6)	ref. group	11 (31.4)	ref. group	10 (23.8)	ref. group	4 (23.5)	ref. group
yes	1225 (50.4)	63 (62.4)	1.6 [1.1-2.4]	24 (68.6)	2.1 [1.0-4.3]	32 (76.2)	3.1 [1.5-6.3]	13 (11.8)	3.1 [1.5-6.3]
unknown	5 (0.2)	0		0		0		0	

ethnic background:									
Norwegian	2265 (93.2)	95 (94.1)	ref. group	30 (85.7)	ref. group	37 (88.1)	ref. group	13 (76.5)	ref. group
western background	90 (3.7)	1 (1.0)	0.3 [0.0-1.9]	2 (5.7)	1.7 [0.4-6.9]	1 (2.4)	0.7 [0.1-4.9]	1 (5.9)	1.9 [0.2-14.6]
non-western background	41 (1.7)	5 (4.9)	2.9 [1.2-6.8]	1 (2.9)	1.8 [0.3-13.2]	3 (7.1)	4.5 [1.4-13.9]	2 (11.8)	8.5 [2.0-36.5]
unknown	34 (1.4)	0		2 (5.7)	4.4 [1.1-17.8]	1 (2.4)	1.8 [0.2-12.7]	1 (5.9)	5.1 [0.7-38.1]

education									
primary school or less	121 (5.0)	6 (5.9)	ref. group	2 (5.7)	ref. group	1 (2.4)	ref. group	1 (5.9)	ref. group
secondary school	903 (37.2)	36 (35.6)	0.8 [0.3-1.9]	16 (45.7)	1.1 [0.3-4.6]	15 (35.7)	2.0 [0.3-15.1]	5 (29.4)	0.7 [0.1-5.7]
high school/university	1401 (57.6)	59 (58.4)	0.8 [0.4-1.9]	17 (48.6)	0.7 [0.2-3.1]	26 (62)	2.2 [0.3-16.4]	11 (64.7)	0.9 [0.1-7.3]
unknown	5 (0.2)	0		0		0		0	

income before tax in 1000 NOK** in 2005									
< 150	670 (27.6)	24 (23.8)	ref. group	12 (34.3)	ref. group	5 (11.9)	ref. group	4 (23.5)	ref. group
150-299	573 (23.6)	22 (21.8)	1.1 [0.6-1.9]	11 (31.4)	1.1 [0.5-2.4]	8 (19.0)	1.9 [0.6-5.7]	4 (23.5)	1.2 [0.3-4.6]
300-500	854 (35.3)	37 (36.6)	1.2 [0.7-2.0]	3 (8.6)	0.2 [0.1-0.7]	18 (42.9)	2.8 [1.0-7.6]	4 (23.5)	0.9 [0.2-3.2]
> 500	323 (13.3)	18 (17.8)	1.5 [0.9-2.8]	8 (22.9)	1.4 [0.6-3.3]	10 (23.8)	4.1 [1.2-12.0]	5 (29.4)	2.6 [0.7-9.6]
unknown	10 (0.4)	0		1 (2.9)	5.6 [0.8-38.9]	1 (2.4)	13.4 [1.7-104.5]	0	

* only those, who reported Chlamydia as a single STI infection in the past 12 months, were included in this group

** NOK - Norwegian crowns

**Table 3:** Sexual behaviour of MSM respondents in the Internet-based cross-sectional study.

Proportion of MSM, reporting Chlamydia, gonorrhoea, HIV infection or syphilis in the previous 12 months and their crude associations									
Sexual behaviour	respondents N = 2430 (%)	Chlamydi a* N= 101 (%)	Crude PR Chlamydia [95% CI]	gonorrhoea N = 35 (%)	Crude PR gonorrhoea [95% CI]	HIV N = 42 (%)	Crude PR HIV [95% CI]	Syphilis N = 17 (%)	Crude PR syphilis [95% CI]
sexual orientation									
homosexual	2088 (85.9)	89 (88.1)	ref. group	30 (85.7)	ref. group	37 (88.1)	ref. group	15 (88.2)	ref. group
bisexual	225 (9.3)	8 (7.9)	0.8 [0.4-1.7]	4 (11.4)	1.2 [0.4-3.5]	3 (7.1)	0.7 [0.2-2.4]	1 (5.9)	0.6 [0.1-4.7]
heterosexual	77 (3.2)	3 (3.0)	0.9 [0.3-2.8]	1 (2.9)	0.9 [0.1-6.5]	2 (4.8)	1.5 [0.4-6.0]	1 (5.9)	1.8 [0.2-13.5]
unsure	40 (1.6)	1 (1.0)	0.6 [0.1-4.1]	0		0		0	

N of male sexual partners in life									
1	68 (2.8)	0		1 (2.9)	ref. group	1 (2.4)	ref. group	1 (5.9)	ref. group
2-5	334 (13.7)	3 (3.0)	ref. group	0		0		0	
6-10	383 (15.8)	6 (5.9)	1.7 [0.4-6.9]	3 (8.6)	0.5 [0.1-5.0]	1 (2.4)	0.2 [0.0-2.8]	0	
11-50	868 (35.7)	40 (39.6)	5.1 [1.6-16.5]	11 (31.4)	0.9 [0.1-6.6]	9 (21.4)	0.7 [0.1-5.5]	5 (29.4)	0.4 [0.0-3.3]
51- > 500	777 (32.0)	52 (51.5)	7.4 [2.3-23.7]	20 (57.1)	1.7 [0.2-12.8]	31 (73.8)	2.7 [0.4-19.6]	11 (64.7)	1.0 [0.1-7.3]

N of male sexual partners in the past 6 months									
0	175 (7.2)	2 (2.0)	ref. group	0		3 (7.1)	ref. group	0	
1-2	978 (40.2)	21 (20.8)	1.9 [0.4-8.1]	6 (17.1)	ref. group	12 (28.6)	0.7 [0.2-2.5]	5 (29.4)	ref. group
3-5	649 (26.7)	29 (28.7)	4.0 [1.0-16.5]	9 (25.7)	2.3 [0.8-6.3]	9 (21.4)	0.8 [0.2-3.0]	4 (23.5)	1.2 [0.3-4.5]
6-10	383 (15.8)	24 (23.8)	5.6 [1.3-23.3]	11 (31.4)	4.7 [1.7-12.6]	6 (14.3)	0.9 [0.2-3.7]	3 (17.6)	1.5 [0.4-6.4]
> 10	242 (10.0)	25 (24.8)	9.2 [2.2-38.3]	9 (25.7)	6.1 [2.2-16.9]	12 (28.6)	2.9 [0.8-10.3]	5 (29.4)	4.4 [1.2-13.8]
unknown	3 (0.1)	0		0		0		0	

anony mous/casual partner in the last 6 months									
no	853 (35.1)	20 (19.8)	ref. group	6 (17.1)	ref. group	12 (28.6)	ref. group	1 (5.9)	ref. group
yes	1572 (64.7)	81 (80.2)	2.2 [1.3-3.6]	29 (82.9)	2.6 [1.1-6.3]	30 (71.4)	1.3 [0.7-2.6]	16 (94.1)	8.7 [1.1-65.3]
unknown	5 (0.2)	0		0		0		0	

UAI** with anonymous/casual partner in the last 6 months									
no	1848 (76.0)	52 (51.5)	ref. group	19 (54.3)	ref. group	24 (57.1)	ref. group	10 (58.8)	ref. group
yes	576 (23.7)	49 (48.5)	3.0 [2.1-4.4]	16 (45.7)	2.7 [1.4-5.2]	18 (42.9)	2.4 [1.3-4.4]	7 (41.2)	2.2 [0.9-5.9]
unknown	6 (0.2)	0		0		0		0	

practice of threesome or group sex									
no	1270 (52.3)	32 (31.7)	ref. group	14 (40.0)	ref. group	13 (30.9)	ref. group	7 (41.2)	ref. group
yes	1068 (43.9)	66 (65.3)	2.4 [1.6-3.7]	21 (60.0)	1.8 [0.9-3.5]	27 (64.3)	2.5 [1.3-4.8]	9 (52.9)	1.6 [0.6-4.1]
unknown	92 (3.8)	3 (3.0)	1.3 [0.4-4.1]	0		2 (4.8)	2.1 [0.5-9.3]	1 (5.9)	2.0 [0.2-15.9]

received money for sex in the last year									
no	2343 (96.4)	93 (92.1)	ref. group	29 (82.9)	ref. group	40 (95.2)	ref. group	16 (94.1)	ref. group
yes	78 (3.2)	7 (6.9)	2.3 [1.1-4.7]	6 (17.1)	6.2 [2.7-14.5]	2 (4.8)	1.5 [0.4-6.1]	1 (5.9)	1.9 [0.2-14.0]
unknown	9 (0.4)	1 (1.0)	2.8 [0.4-17.9]	0		0		0	

paid for sex in the last year									
no	2305 (94.9)	90 (89.1)	ref. group	29 (82.9)	ref. group	39 (92.9)	ref. group	16 (94.1)	ref. group
yes	118 (4.9)	11 (10.9)	2.4 [1.3-4.3]	5 (14.3)	3.4 [1.3-8.5]	3 (7.1)	1.5 [0.5-4.8]	1 (5.9)	1.2 [0.2-9.1]
unknown	7 (0.3)	0		1 (2.9)	11.3 [1.8-72.2]	0		0	

how many times feeling drunk in a given month									
< 1	487 (20.0)	8 (7.9)	ref. group	2 (5.7)	ref. group	10 (23.8)	ref. group	4 (23.5)	ref. group
1	416 (17.1)	14 (13.9)	2.0 [0.9-4.8]	9 (25.7)	5.3 [1.1-24.2]	12 (28.6)	1.4 [0.6-3.2]	4 (23.5)	1.2 [0.3-4.6]
2-3	588 (24.2)	27 (26.7)	2.8 [1.3-6.1]	12 (34.3)	5.0 [1.1-22.1]	12 (28.6)	1.0 [0.4-2.3]	3 (17.6)	0.6 [0.1-2.8]
≥ 4	923 (38.0)	52 (51.5)	3.4 [1.6-7.2]	12 (34.3)	3.2 [0.7-14.1]	8 (19.0)	0.4 [0.2-1.1]	5 (29.4)	0.6 [0.2-2.4]
unknown	16 (0.7)	0		0		0		1 (5.9)	7.6 [0.9-64.3]

unsafe sex under the influence of alcohol in the past 12 months									
no	1729 (71.1)	49 (48.5)	ref. group	18 (51.4)	ref. group	27 (64.3)	ref. group	11 (64.7)	ref. group
yes	641 (26.4)	51 (50.5)	2.8 [1.9-4.1]	17 (48.6)	2.5 [1.3-4.9]	15 (35.7)	1.5 [0.8-2.8]	6 (35.3)	1.5 [0.5-4.0]
unknown	60 (2.5)	1 (1.0)	0.6 [0.1-4.2]	0		0		0	

under the influence of selected drugs*** during sex in the past 12 months									
no	2106 (86.7)	81 (83.2)	ref. group	25 (71.4)	ref. group	25 (59.5)	ref. group	14 (82.3)	ref. group
yes	240 (9.9)	17 (16.8)	1.8 [1.1-3.0]	10 (28.6)	3.6 [1.8-7.5]	16 (38.1)	5.6 [3.0-10.4]	3 (17.6)	1.9 [0.5-6.5]
unknown	84 (3.5)	3 (3.0)	0.9 [0.3-2.9]	0		1 (2.4)	1.0 [0.1-7.3]	0	

current steady relationship with a woman									
no	2113 (86.9)	89 (88.1)	ref. group	28 (80.0)	ref. group	36 (85.7)	ref. group	13 (76.5)	ref. group
yes	236 (9.7)	10 (9.9)	1.0 [0.5-1.9]	4 (11.4)	1.2 [0.4-3.6]	5 (11.9)	1.2 [0.5-3.1]	3 (17.6)	2.1 [0.6-7.2]
unknown	81 (3.3)	2 (2.0)	0.6 [0.1-2.3]	3 (8.6)	2.8 [0.9-9.0]	1 (2.4)	0.7 [0.1-5.2]	1(5.9)	2.0 [0.3-15.1]

current steady relationship with a man									
no	1492 (61.4)	65 (64.4)	ref. group	19 (54.3)	ref. group	26 (61.9)	ref. group	8 (47.1)	ref. group
yes	936 (38.5)	36 (35.6)	0.9 [0.6-1.3]	16 (46.7)	1.3 [0.7-2.6]	16 (38.1)	1.0 [0.5-1.8]	9 (52.9)	1.8 [0.7-4.6]
unknown	2 (0.1)	0		0		0		0	

ever had a date in reality with an Internet partner									
no	190 (7.8)	3 (3.0)	ref. group	0	no variance	2 (4.8)	ref. group	1 (5.9)	ref. group
yes	2209 (90.9)	97 (96.0)	2.8 [0.9-8.7]	35 (100)		40 (95.2)	1.7 [0.4-7.1]	16 (94.1)	1.4 [0.2-10.3]
unknown	31 (1.3)	1 (1.0)	2.0 [0.2-19.0]	0		0		0	

* only those, who reported Chlamydia as a single STI infection in the past 12 months, were included in this group

**UAI - unprotected anal intercourse

***selected drugs included: marihuana, prescription drugs, ecstasy, LSD, GHB, cocaine, heroine, amphetamines, methamphetamines

**Table 4:** Associations of potential risk factors with three different STI outcomes among MSM respondents of the Internet-based cross-sectional study.

Results represent three separate multivariable models with self-reported STI as outcomes. Different exclusion criteria were applied in these models, as described in the text and presented in the Table 1.						
Potential risk factors	Chlamydia N = 101		gonorrhoea N = 35		HIV N = 42	
	
	Adjusted PR [95% CI]	p for trend	Adjusted PR [95% CI]	p for trend	Adjusted PR [95% CI]	p for trend
age groups (years)		0.002		0.255		0.932
16-25	ref. group		ref. group		ref. group	
26-35	0.6 [0.4-1.1]		0.5 [0.2-1.6]		1.4 [0.6-3.2]	
36-45	0.4 [0.2-0.9]		0.7 [0.2-2.3]		0.8 [0.3-2.1]	
≥ 46	0.3 [0.1-0.7]		0.6 [0.1-2.2]		1.5 [0.6-3.8]	

residing in Oslo or Akershus county						
no	ref. group		ref. group		ref. group	
yes	1.4 [1.0-2.1]		1.9 [0.9-4.1]		2.3 [1.2-4.6]	

ethnic background:						
Norwegian	ref. group		ref. group		ref. group	
immigrant with western background	0.3 [0.0-1.8]		1.5 [0.4-6.1]		0.4 [0.0-3.0]	
immigrant with non-western background	2.8 [1.4-5.7]		1.8 [0.5-6.1]		5.4 [1.9-15.3]	
unknown	not included		5.9 [1.3-26.3]		not included	

education		0.568		0.987		not
primary school or less	ref. group		ref. group		merged with ref. group	calculated
secondary school	0.6 [0.3-1.2]		1.7 [0.2-16.1]		ref. group	
high school/university	0.5 [0.3-1.1]		1.6 [0.1-16.5]		0.8 [0.5-1.5]	

annual income before tax in 1000 NOK* in 2005		0.204		0.380		0.102
< 150	ref. group		ref. group		ref. group	
150-299	1.1 [0.6-1.9]		1.0 [0.4-2.5]		1.0 [0.4-2.7]	
300-500	1.2 [0.6-2.1]		0.1 [0.0-0.6]		0.8 [0.3-1.9]	
> 500	1.3 [0.6-2.9]		1.0 [0.3-2.9]		1.5 [0.6-4.3]	
unknown	not included		not included		10.4 [1.5-71.4]	

N of male sexual partners in life:		< 0.001		0.056		< 0.001
1-10	ref. group		ref. group		ref. group	
11-50	4.0 [1.8-8.8]		2.4 [0.7-7.6]		3.0 [0.7-13.2]	
51- > 500	4.4 [1.9-10.3]		4.5 [1.3-15.6]		13.9 [3.5-55.8]	

N of male sexual partners in the past 6 months		0.308		0.065		0.544
0-2	ref. group		ref. group		ref. group	
3-5	1.2 [0.6-2.2]		1.7 [0.6-4.9]		0.6 [0.2-1.8]	
6-10	1.2 [0.7-2.3]		3.1 [1.1-8.8]		0.4 [0.1-1.5]	
> 10	1.6 [0.8-3.2]		3.4 [1.0-11.3]		0.7 [0.2-2.3]	

practice of threesome or group sex						
no	ref. group		ref. group		ref. group	
yes	1.5 [1.0-2.3]		0.8 [0.4-1.7]		1.0 [0.5-2.1]	

received money for sex in the last year						
no	ref. group		ref. group		ref. group	
yes	0.9 [0.3-2.2]		2.3 [0.7-8.4]		1.9 [0.4-8.3]	

paid for sex in the last year						
no	ref. group		ref. group		ref. group	
yes	1.6 [0.8-3.2]		1.2 [0.3-3.9]		0.6 [0.1-2.2]	
unknown	not included		5.5 [0.4-82.7]		not included	

how many times feeling drunk in a given month		0.409		0.283		< 0.001
< 1	ref. group		ref. group		ref. group	
1	1.6 [0.7-4.0]		3.9 [0.9-16.8]		1.1 [0.4-2.7]	
2-3	2.0 [0.9-4.5]		3.3 [0.8-12.9]		0.6 [0.2-1.3]	
≥ 4	1.6 [0.7-3.5]		1.3 [0.3-5.0]		0.1 [0.0-0.3]	

unsafe sex under the influence of alcohol in the past 12 months						
no	ref. group		ref. group		ref. group	
yes	1.8 [1.1-2.9]		1.9 [0.9-4.3]		1.5 [0.7-3.4]	

under the influence of selected drugs** during sex in the past 12 months						
no	ref. group		ref. group		ref. group	
yes	0.8 [0.5-1.5]		1.7 [0.8-3.9]		5.2 [2.7-11.4]	

ever had a date in reality with Internet partner						
no	ref. group		not included		ref. group	
yes	1.6 [0.5-5.0]				1.4 [0.4-4.6]	

UAI*** with a casual or anonymous partner in the last 6 months						
no	ref. group		ref. group		ref. group	
yes	1.5 [0.9-2.4]		1.0 [0.5-2.2]		2.0 [0.9-4.6]	

* NOK - Norwegian crowns

**selected drugs included: marihuana, prescription drugs, ecstasy, LSD, GHB, cocaine, heroine, amphetamines, methamphetamines

*** UAI - unprotected anal intercourse

### Statistical analyses

We used Stata 9.0 for the analyses. Associations between reported recent selected STI and categorical independent variables with and without adjustment for potential confounders were studied by Poisson regression with robust variance estimates [13]. Crude and adjusted prevalence ratios (PR) with 95% confidence intervals (CI) were calculated. Three separate multivariable models were generated (Table 1) and they included variables, associated with the outcome at p < 0.15 [14] in crude analysis as well as variables, previously shown as predictors of STI. Where the unknown or missing were statistically different in the crude analysis from the rest of the categories within a variable, they were entered in multivariable analysis as a separate category, as presented in the Table 4, otherwise they were excluded. The variable "ever had a date in reality with a partner from the Internet" was not included in the multivariable analysis with gonorrhoea as an outcome due to a zero variance of this potential predictor. Variables health region and residing in Oslo/Akershus were created from the same original variable and, due to our study question, only the latter was selected for multivariable analysis.

In each multivariable model, we excluded those, who did not report an outcome of interest, but were infected with another selected STI - as presented in the Table 1. In addition, we also excluded those, reporting Chlamydia and selected STI in the past 12 months, from the model, where Chlamydia was a sole outcome. Those with another STI were included in the outcomes of the other two corresponding models to represent more at risk populations.

Participation in the study was of no direct benefit to the respondents. The study was approved by the Regional Committee for Medical and Health Research Ethics, Southern Norway, and by the Norwegian Data Inspectorate.

## Results

Out of 2598 respondents, 2430 were included in the study after application of our exclusion criteria. The median age of respondents was 31 years, ranging from 16 to 74 years. The respondents were from all regions of Norway, although half of them lived in Oslo or neighbouring Akershus county (Table 2). Most respondents reported being ethnic Norwegians (93%) and having university education (58%). Most men stated their sexual orientation as homosexual (86%), 68% reported more than 10 male sexual partners in their life and 65% reported having had intercourse with an anonymous or casual partner in the last 6 months, of which more than a third reported having unprotected anal intercourse (UAI), (Table 3). We identified 184 (8%) MSM, infected with a selected STI in the past 12 months: 17 (0.7%) reported being diagnosed with syphilis, 35 (1.4%) with gonorrhoea, 42 (1.7%) with HIV and 126 (5.2%) with Chlamydia. In addition, 26 (1.1%) respondents reported being diagnosed with HIV prior to the past twelve months.

Having unsafe sex in the past 12 months under the influence of alcohol was reported by 26.4%. Those who reported feeling drunk four times and more in an average month, represented 38.0% and those who used selected drugs (marihuana, prescription drugs, ecstasy, LSD, GHB, cocaine, heroine, amphetamines or methamphetamines) in connection to sex represented 9.9% of the participants (Table 3).

Crude analyses indicated no associations between a selected STI and age, with the exception of those above 45 years, who were less likely to report Chlamydia (Table 2). Compared to those in health region East (which includes Oslo), those living in health region West in Norway had a lower prevalence ratio for HIV infection and those in the North for Chlamydia. In crude analysis, residing in Oslo or Akershus county was associated with all selected STI. Those with non-western background were more likely to be recently diagnosed with Chlamydia, HIV and syphilis; and those, who did not want to reveal their ethnic background, with gonorrhoea. Education did not seem to be associated with any selected STI in crude analysis, while yearly income, higher than 300 000 Norwegian crowns, as well as unrevealed income, seemed to increase the prevalence ratio for HIV. Conversely, an income of 300-500 thousand Norwegian crowns per year seemed to be protective for gonorrhoea (Table 2).

Among possible sexual exposures, having more than 10 male partners in life was associated with Chlamydia in crude analysis, but it did not seem to be important for other selected STI (Table 3). Number of partners in the past six months was, on the other hand, more important for Chlamydia, gonorrhoea and syphilis, but not for the HIV infected. Selected STI were not associated with sexual orientation, choosing partner on the Internet and steady relationship with either a man or a woman, while many other risk behaviours were associated with all or at least two of the selected STI (Table 3).

In the multivariable model, a decreasing linear trend between Chlamydia and age was observed (p for trend = 0.002), (Table 4). Compared to those without selected STI, HIV infection was more prevalent among residents of Oslo or Akershus county, while the results on residence were inconclusive for gonorrhoea (p = 0.082) and Chlamydia (p = 0.068). Immigrants with non-western background were more likely to report HIV and Chlamydia infection. While unrevealed income was associated with HIV, income did not seem to be relevant for Chlamydia and was, in the category of 300-500 thousand Norwegian crowns per year, protective for gonorrhoea. A positive linear trend between the number of male sexual partners in life and the prevalence of Chlamydia and HIV was observed (p for trend < 0.001), while only the category above 50 lifetime partners was associated with gonorrhoea. Number of male sexual partners in the past six months was not important correlate for Chlamydia and HIV, but was, above counts of 5, important for gonorrhoea. The practice of group sex could be a potential risk factor for Chlamydia (p = 0.069). Receiving money or paying for sex was not important for any of the outcomes. The frequency of feeling drunk in an average month was inversely associated with HIV (p for trend < 0.001), while reporting unsafe sex under the influence of alcohol in the past 12 months was correlated to Chlamydia. Being under the influence of selected drugs during sex in the past 12 months was associated with HIV infection. Results for having a date with a partner whom they met on the Internet, and UAI with a casual or anonymous partner in the last 6 months, were inconclusive.

## Discussion

This is the first Internet study on sexual risk behaviour among MSM in Norway. Our predominantly well-educated study population frequently used the Internet for dating, reported prevalent partner exchange including recent casual or anonymous partners, and alcohol use. MSM, being diagnosed with Chlamydia, HIV, gonorrhoea or syphilis in the past year represented 8% of our respondents.

Our results suggest that MSM, who reported any selected STI in the past year, represent different demographic groups and groups with different risk behaviours. Younger age, non-western background, number of lifetime male sexual partners and unsafe sex under the influence of alcohol in the past 12 months were factors associated with Chlamydia. Similarly, non-western background was also associated with HIV infection, as well as residence in Oslo or Akershus county, unrevealed income, more than 50 lifetime male sexual partners and being under the influence of selected drugs during sex in the past 12 months. HIV infection was decreasingly associated with the frequency of feeling drunk in a given month. Gonorrhoea was associated with unrevealed ethnic background, more than 50 lifetime male sexual partners and having more than 5 male sexual partners in the past 6 months. Reporting a mid-range income category seemed to be protective.

Collecting data with no human interviewers and without any personally identifying information might have been grounds for more revealing answers on behaviour. Our study has received considerable public attention and was well-known among MSM. Thus, we were able to collect data from relatively large numbers of respondents from all health regions in Norway. High Internet coverage and almost universal computer literacy in Norway made our study widely accessible. Since our questionnaire took about 45 minutes to complete, we assume double entries were rare.

Representativeness and generalization of the results to the entire MSM population in Norway might not be feasible, as Internet sampling is subject to selection bias, misrepresentation as a member of the sampled population, repeated participation, missing data, inability to gather biological specimens etc. [9]. Those with an STI in the past year might have been more likely to be aware of the past risk behaviour (recall bias) - thus overestimating the effect size - and to respond as we posted the banner inviting respondents to "help prevent HIV infection" (selection bias). Using self-reporting to estimate STI prevalence could introduce measurement error. The prevalences of self-reported STI in the past year are likely to be an underestimation in our study, as some MSM might not be aware of their (sometimes asymptomatic) current infection or refuse to be tested [15], which could decrease the associations, found in our study. Similarly, respondents with an STI, which was not selected as an outcome, could also decrease these associations. To estimate the frequency of alcohol consumption, we used a rather subjective "feeling of being drunk". We did not specifically ask whether some drugs were injected, however such HIV transmission is rare in Norway due to effective harm reduction programmes: in total, 10 men were reported being infected with HIV due to injecting drugs in Norwegian surveillance system for communicable diseases in 2007 and 7 in 2006 [4]. Since no adjustment for multiple comparisons was made, some of the significant associations might appear due to chance. Despite the large number of participants, we were not able to show statistically significant effects for rare exposures (such as not having a date with Internet partner and paying or receiving money for sex), when the effect size is small. Gonorrhoea as an outcome was rare, which limited our power to detect factors associated with infection. Nonetheless, we believe the study provides an important insight into current MSM behaviour in Norway.

Comparing the findings of our study with other studies is of limited value, as there are notable differences in recruitment sites, inclusion criteria, methodology (including definitions) and STI epidemiology among MSM.

An Internet study from USA, focusing on a six-month period in 2001, reported 0.3% being newly diagnosed with syphilis, 1.8% with gonorrhoea and 1.1% with Chlamydia; while total HIV prevalence of the participants, including non-recent infections, was 7.2% [16], similar to a Danish study from 2006 with 8% [17].

More than a half (57.6%) of our respondents reported finishing high school or university, which was high compared to corresponding Norwegian male population (24.5% in January 2008) [12], but similar findings (55%) were reported from Danish study recruiting MSM at different venues and online [17]. Immigrants with non-western background seemed underrepresented in our sample (1.7% compared to 6.3% males in Norwegian population [18]); perhaps due to language, cultural barriers or lower proportion of self-identified MSM among them.

A case-control study of MSM from Chicago and Los Angeles found recent HIV seroconversion to be associated with low income, UAI with HIV positive partners, and using Viagra and poppers [19]. In a longitudinal American study, HIV acquisition was found to be related to ethnicity (Black race), use of alcohol or drugs before sex, receptive UAI, insertive UAI with HIV positive partners, use of alcohol or drugs before sex, reporting 4 or more male partners in the last 6 months, amphetamines and heavy alcohol use [20].

In a prospective study in Australia, urethral gonorrhoea and Chlamydia in MSM were associated with these common risk factors: younger age, contact with gonorrhoea or Chlamydia infected sexual partner and a higher number of casual partners in the past 6 months. In addition, gonorrhoea was associated with UAI with HIV positive casual partners, and urethral Chlamydia with more frequent insertive oral sex with ejaculation with casual partners. When no receptive UAI was reported, anal infections with Chlamydia and gonorrhoea were associated with a variety of non-intercourse-receptive anal practices with casual partners [21].

MSM were previously identified as a population with high prevalence of alcohol use [22]. A review article by Woolf and Maisto concluded heavy episodic drinking among MSM is related to HIV infection, while history or frequency of consumption might not be [22].

Our results suggest Norwegian MSM share some similarities in risk behaviour with MSM around the world, perhaps due to sharing popular culture and ideas on the Internet and an increase in international travel [3].

Chlamydia, syphilis and HIV infection may be present for a long time before being noticed or diagnosed, contributing to the fact that relevant exposures, leading to the infection, might have happened a long time ago and behaviour might have changed during this time or, particularly, after the diagnosis and counselling. We can see that some potential risk factors, limited to the past 6 or 12 months before the study, were not important for these infections. Number of lifetime male sexual partners seemed to be more important for HIV and Chlamydia than number of male sexual partners in the past 6 months. In addition, pharyngeal infections result from oral sex practices, potentially decreasing the importance of UAI in transmission of Chlamydia [23], gonorrhoea and syphilis.

The inverse association of Chlamydia and age was expected due to diminishing testing activity and prevalence after age 40 [7]. Chlamydia is geographically-widespread infection in Norway [24] and clustering of MSM cases in Oslo or Akershus, as previously described for gonorrhoea and syphilis [5,6] and as suggested also for HIV infection, might be less emphasized. Nonetheless, most MSM in our study do come from Oslo or Akershus and engaging in sex with multiple and casual partners is likely adjacent to the urban lifestyle.

Although reported by only 1.7% of MSM in our study, non-western background was consistently identified as a risk factor for Chlamydia and HIV. Immigrants from areas with a generalised HIV/STI epidemic could be more likely to know their status as they are offered HIV testing upon arrival to Norway; but these were not likely to answer our questionnaire as it was in Norwegian. Specific reasons for vulnerability of immigrant MSM for STI in Norway could be a subject of further research.

Alcohol might influence the STI transmission by behaviour, sexual arousal, adverse effects on the immune system or perhaps a third, confounding variable (e.g., "sensation-seeking") [25]. MSM may drink to achieve a "cognitive escape", for example to avoid "being worried about HIV/AIDS" [22,26]. Although not significant, prevalence ratio estimates were highest among "moderate drinkers" (groups feeling drunk once or up to three times in an average month) among gonorrhoea and Chlamydia cases. This might imply drinking in social situations and venues (e.g., bars), connected to sensation- and partner-seeking [22]. Our reverse association between the increasing frequency of feeling under the influence of alcohol and HIV infection is puzzling; however drugs seem to be a more important correlate than alcohol in this group. The importance of alcohol in STI transmission is on the other hand emphasized by "unsafe sex under the influence of alcohol in the past 12 months" associated with higher prevalence ratio for Chlamydia. Thus, alcohol use in sexual risk behaviour remains "a controversial topic with mixed findings" [27].

Being under the influence of selected drugs during sex was associated with reported HIV infection in the last year, but not with Chlamydia or gonorrhoea. Drugs may be used before or during sex to enhance sexual pleasure, but their use may have complex and harmful physiological or cognitive side-effects, enhancing the likelihood of frequent partner change and unprotected anal sex with HIV positive partners or partners of unknown serostatus [19]. Further research is needed on the importance of specific drugs in HIV transmission among MSM in Norway.

## Conclusions

This first Internet study on sexual risk behaviour of MSM in Norway has reached a large and active online MSM community, thus the possibility of Internet based health interventions could be further explored. Our study demonstrates different associations of demographic and behavioural factors with different STI outcomes in the study population. The number of male sexual partners and ethnic background seem to be the most important predictors for Chlamydia, gonorrhoea and HIV. Additional research is needed to analyse the association of STI with specific drug and alcohol use. To evaluate time trends and the effectiveness of preventive measures, behavioural studies among MSM in Norway should be repeated regularly.

## Competing interests

The authors declare that they have no competing interests.

## Authors' contributions

IJ drafted the manuscript. BS, EK and PA took part in the planning of the study. BS helped with data collection. IJ, AMG, BS, EK and PA contributed to the study design, analysis and interpretation. All authors critically reviewed and approved the final version of this paper for publication. PA is the guarantor.

## Pre-publication history

The pre-publication history for this paper can be accessed here:

http://www.biomedcentral.com/1471-2334/10/261/prepub
